# Higher Drop in Speed during a Repeated Sprint Test in Soccer Players Reporting Former Hamstring Strain Injury

**DOI:** 10.3389/fphys.2017.00025

**Published:** 2017-01-27

**Authors:** Ola D. Røksund, Morten Kristoffersen, Bård E. Bogen, Alexander Wisnes, Merete S. Engeseth, Ann-Kristin Nilsen, Vegard V. Iversen, Silje Mæland, Hilde Gundersen

**Affiliations:** ^1^Department of Occupational Therapy, Physiotherapy and Radiography, Bergen University CollegeBergen, Norway; ^2^Department of Sport and Physical Activity, Bergen University CollegeBergen, Norway; ^3^The Norwegian Olympic Sports Center (Olympiatoppen-Vest)Bergen, Norway; ^4^Uni Research HealthBergen, Norway

**Keywords:** hamstring strain, repeated sprint, fatigue, soccer players

## Abstract

**Aim:** Hamstring strain injury is common in soccer. The aim of this study was to evaluate the physical capacity of players who have and have not suffered from hamstring strain injury in a sample of semi-professional and professional Norwegian soccer players in order to evaluate characteristics and to identify possible indications of insufficient rehabilitation.

**Method:** Seventy-five semi-professional and professional soccer players (19 ± 3 years) playing at the second and third level in the Norwegian league participated in the study. All players answered a questionnaire, including one question about hamstring strain injury (yes/no) during the previous 2 years. They also performed a 40 m maximal sprint test, a repeated sprint test (8 × 20 m), a countermovement jump, a maximal oxygen consumption (VO_2max_) test, strength tests and flexibility tests. Independent sample *t*-tests were used to evaluate differences in the physical capacity of the players who had suffered from hamstring strain injury and those who had not. Mixed between-within subject's analyses of variance was used to compare changes in speed during the repeated sprint test between groups.

**Results:** Players who reported hamstring strain injury during the previous two years (16%) had a significantly higher drop in speed (0.07 vs. 0.02 s, *p* = 0.007) during the repeated sprint test, compared to players reporting no previous hamstring strain injury. In addition, there was a significant interaction (groups × time) (*F* = 3.22, *p* = 0.002), showing that speed in the two groups changed differently during the repeated sprint test. There were no significant differences in relations to age, weight, height, body fat, linear speed, countermovement jump height, leg strength, VO_2max_, or hamstring flexibility between the groups.

**Conclusion:** Soccer players who reported hamstring strain injury during the previous 2 years showed significant higher drop in speed during the repeated sprint test compared to players with no hamstring strain injury. The maximal speed, leg strength, ability to produce maximal power, endurance capacity, and hamstring flexibility was similar for both groups. Thus, a repeated sprint test consisting of 8 × 20 m could be used as a field-based diagnostic tool to identify players in need of reconditioning programs to ensure complete post-injury rehabilitation.

## Introduction

Hamstrings strain injuries are common among soccer players, and account for 12–16% of all soccer-related injuries (Arnason et al., 2004; Woods et al., 2004; Ekstrand et al., 2011a; Hagglund et al., 2016). Although there has been a great focus on prevention and treatment of these injuries, the relative number of registered cases has not decreased over time (Ekstrand et al., 2011a,b, 2016). The consequences of hamstring strain injury depends largely on the severity, and the extent of the damage can vary from microscopic muscle-fiber ruptures to a complete tear involving several muscle fascicles (Opar et al., 2012). These injuries account for more than one-third of all time-loss in high-level European professional football, and represent a significant cost burden for the clubs and a major challenge to the players' careers (Woods et al., 2004; Ekstrand et al., 2016).

It is important for both clubs and players that rehabilitation and return to play takes place as quickly as possible after hamstring strain injury. On the other hand, incomplete rehabilitation increases the risk of another and often more severe hamstring strain injury (Maniar et al., 2016). Since the most commonly cited risk factor for a future hamstring strain injury is a previous hamstring strain injury (Arnason et al., 2004; Hägglund et al., 2006), sufficient rehabilitation is crucial. To prevent re-injury, it is important to identify the physical capacities that can identify insufficient rehabilitation. In previous studies, various measurements of complete rehabilitation after a hamstring strain injury such as leg strength, hamstring flexibility (Maniar et al., 2016), and sprinting speed have been used (van der Horst et al., 2016). However, there is to date no gold standard, and the recurrence rate of hamstring strain injury is reported to be as high as 33% (van der Horst et al., 2016).

Endurance, acceleration, deceleration, maximal sprinting and repeated sprinting ability are important capacities in soccer (Ingebrigtsen et al., 2015). The hamstring muscle is very important in sprint acceleration performance and in maximal sprinting (Morin et al., 2015). The vast majority of hamstring strain injuries in soccer occur when running (Woods et al., 2004; Opar et al., 2012), most often late in the match or training session (Woods et al., 2004; Mohr et al., 2005; Ekstrand et al., 2011a), which suggests that neuromuscular fatigue plays a role (Small et al., 2009; Russell et al., 2016). Thus, there is a need of a specific test, with ecological validity, able to identify those players standing into a risk of hamstring re-injury. Most rehabilitation tests are performed maximal effort without muscular fatigue. One exception was a recent study which showed increased neuromuscular fatigue, measured as reduced jump height after repeated sprints, in players with former lower-limb injury (Padulo et al., 2016).

In the present study, the aim was to evaluate the physical capacities of players who had and had not suffered from hamstring strain injuries in a sample of semi-professional and professional Norwegian soccer players in order to identify possible indications of insufficient rehabilitation.

## Methods

### Design

**Figure 1:**
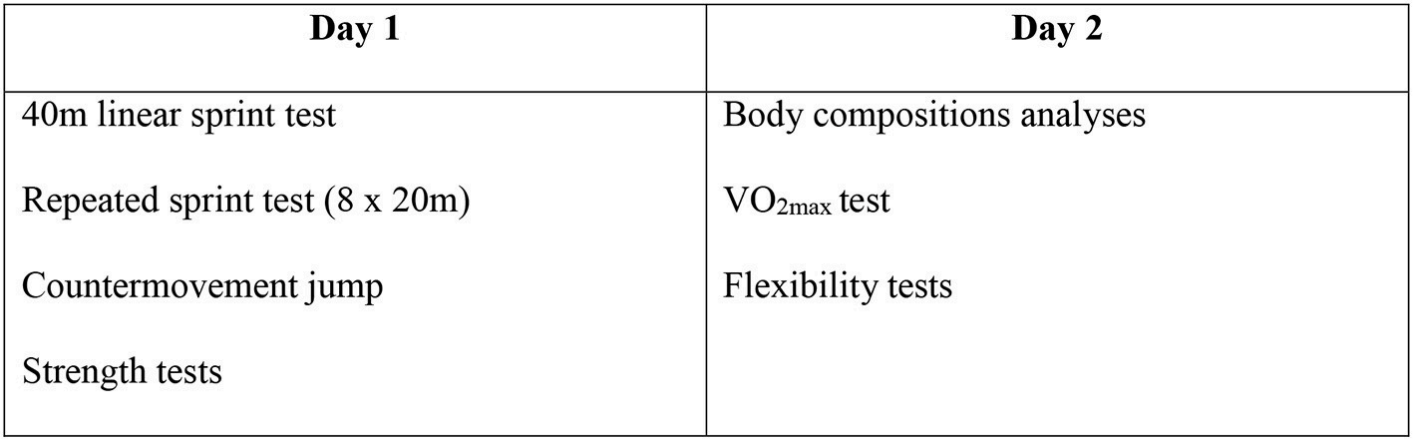
**An overview of the test orders**.

A cross-sectional design was employed, with data collection from January to March 2015. All physical tests were performed during 2 subsequent days for each player. Sprinting tests were performed in an indoor area with a Dynaspike running surface, whereas countermovement jump, strength, flexibility, and maximal oxygen consumption (VO_2max_) tests were conducted in the physiological testing laboratory at Bergen University College (Figure 1).

### Participants

**Figure 2:**
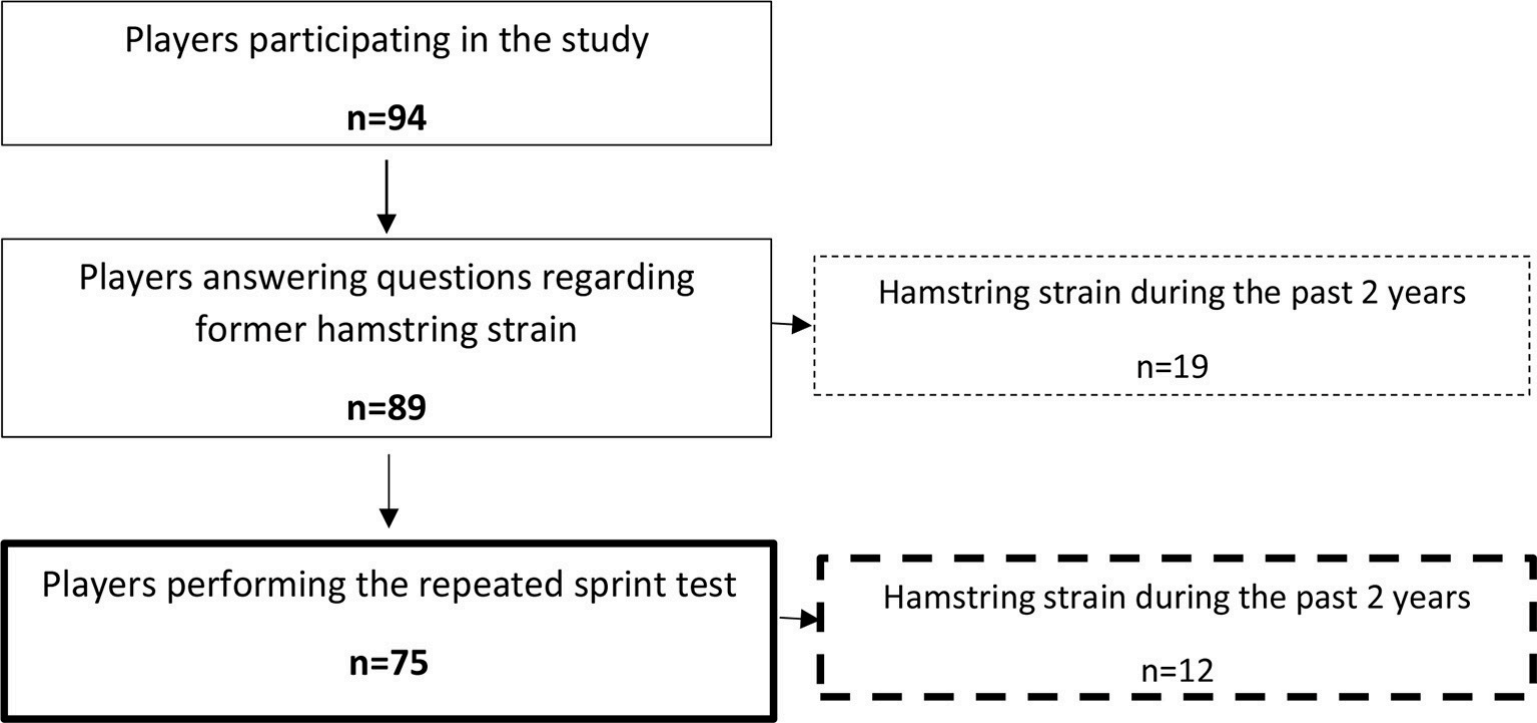
**An overview of the participating players**. Only players answering questions regarding former hamstring strain injury and performing the repeated sprint test, the 40 m linear sprint test and the hamstring flexibility test were included in the final analyses.

In total, 94 male semi-professional and professional soccer players (20 ± 4 years, 75 ± 10 kg, 181 ± 6 cm) who play at the second and third level in the Norwegian league participated in the study, which included a questionnaire and a variety of physical tests (Figure 1). Eighty-nine of the players answered the question regarding previous hamstring strain injury, and 75 of the players performed the repeated sprint test (Figure 2).

### Procedures

Players were instructed not to engage in strenuous exercise the day before testing, and to perform all tests in t-shirt, shorts, and suitable shoes. They were also told to eat and drink properly before testing, and to drink and eat a light meal containing carbohydrates between the sprint and the strength tests.

#### The 40 m linear sprint test

Before the sprint tests, all players went through a standardized 15-min warm-up protocol led by a physical trainer. The warm-up protocol consisted of a 10-min general part with low intensity running. Then, players performed 5-min of guided stretching (i.e., of hamstrings, quadriceps, iliopsoas, and hip adductors). Finally, players performed 4 × 40–50 m linear runs with increasing intensity and speed, followed by two maximal linear accelerations of 20 mm.

After the warm-up, all players performed three maximal sprints of 40 m separated by 2–3 min of rest. Players started in a standing start position with split legs, and the toes of the front foot placed on the starting line 50 cm behind the first photogates. The players started when ready. They were not allowed to move their body backward before starting. The same starting procedures were followed for the repeated sprint test.

Times were recorded using Brower equipment (Wireless Sprint System, USA, accuracy of 0.01 s). The height of the first photogates was 40 cm to avoid the hand of the player to start the timer, whereas the photogates at 40 m were 60 cm high. Only the best attempt was included in the analyses.

#### The 8 × 20 m repeated sprint test

After a 10-min rest, players performed the repeated sprint test, which consisted of 8 × 20 m sprint starting every 30 s. Players were instructed to perform every sprint with maximal effort. Times at 0–10 m (T_10_), 0–20 m (T_20_), and 10–20 m (T_10−20_) were recorded using Brower equipment (Wireless Sprint System, USA). The mean time for all eight runs, and the drop in speed [(mean time of seventh and eighth runs) minus (mean time of first and second runs)] was calculated (Psotta et al., 2005) to evaluate the ability to maintain maximal speed during the test.

#### Counter movement jump (CMJ)

The counter movement jump (CMJ) test was performed using a Kistler 9286B force plate (Kistler Instruments AG, Winterthur, Switzerland). From a standing position (with hands on hips and a knee angle of 180°), a countermovement was performed (until a knee angle of ~90°), followed by an immediate jump. The jumping height was calculated from take-off velocity (Dello Iacono et al., 2016) using the Kistler MARS software. The best of three attempts was used in the analyses.

#### Leg strength

A five repetition maximum (RM) back squat test was used to determine leg strength. The players started in an up-right position, with parallel feet hip-width apart, and with an external weight braced across the trapezius muscle or rear deltoid muscle in the upper back. The back squat test started with a flexion of the hip and knee joints. In the lowest position, the hip joints had to be lower than the knee joints. The hip and knee joints were fully extended between each repetition. The negative and the positive phases of the lift took ~2 s each. A standardized warm-up protocol with increasing weight (10 repetitions with 20 kg, 8 × 40 kg, 8 × 50 kg, 5 × 60 kg) was followed for all players. After the warm-up players increased weight until 5 RM was reached. The technique was supervised and approved by a physical trainer.

#### Body composition

A direct segmental multi-frequency bioelectrical impedance analysis (DSM-BIA) for determining body composition was performed using the In-Body720 (Biospace Co. Ltd, Seoul, Korea) body composition analyzer (Lim et al., 2009; Ling et al., 2011; Tompuri et al., 2015). Standard procedures were followed for all players. Body weight (kg) and body fat (%) were used in the statistical analyses.

#### Maximal oxygen consumption

Gas exchange values were measured using an Oxycon Pro apparatus (Jaeger GmbH, Hoechberg, Germany) with a mixing chamber. The flowmeter was calibrated with a 3 L volume syringe (Hans Rudolph Inc., Kansas City, MO). Before each measurement, the volume of oxygen (VO_2_) and volume of carbon dioxide (VCO_2_) gas analyzers were calibrated using high-precision gases (16.00 ± 0.04% O_2_ and 5.00 ± 0.1% CO_2_, Riessner-Gase GmbH & co, Lichtenfels, Germany). Heart rate (HR) was measured with an HR monitor (Polar V800, Polar Electro OY, Kempele, Finland), using a 5 s interval for data storage. VO_2max_ was defined as the highest 1 min average VO_2_ during the test. Maximal HR was defined as the highest value that was attained on average over a 5 s period at the final stage of the protocol. VO_2_ consumption, respiratory exchange ratio (RER), volume of expired air (VE), velocity, and HR were recorded and stored during the tests, whereas blood lactate concentration was measured by taking samples from the fingertip using a Biosen S-line (EKF diagnostics, Germany).

The test started with a 10-min warm up, running at 10 km·h^−1^ at an inclination of 1.7% on a motorized treadmill (Woodway PPS 55, USA). VO_2max_ was determined using an incremental running test. The test began at 11 km·h^−1^ at an inclination of 5.2%, and the velocity was increased by 1 km·h^−1^ every 1 min until exhaustion. Exhaustion was defined as meeting three of the following four criteria (Bassett and Howley, 2000): (1) HR within five beats of the participants self-reported HR_max_; (2) Lactate level above 8.0 mmol/l; (3) RER > 1.15; (4) VO_2_ consumption plateau, or decrease in VO_2_ consumption with increasing work rate.

#### Hamstring flexibility

Hamstring flexibility was measured using the sit and reach test (Wells and Dillon, 1952; The Canadian Physical Activity Fitness and Lifestyle Approach (CPAFLA), 2003) and the straight leg raise test (Kendall and McCreary, 2005). Two experienced physiotherapists supervised all of the tests, following standardized test procedures. In the sit and reach test, the player sat on the floor with knees extended. Shoes were removed and the soles of the feet were placed flat against a standardized box developed for the sit and reach test. The player slowly reached forward with parallel arms, placing his hands as far as possible forward on the box in front of his feet. With the help of a ruler placed on the top of the box (scale 3), the player's flexibility was measured in cm. The player was instructed to reach as far as possible and to hold the end position for 2 s. The test has been recommended to measure hamstring flexibility (Mayorga-Vega et al., 2014).

In the straight raise leg test, the player was in a supine start position. One leg was passively moved into hip flexion until a firm end-feel was ascertained. The knee of the moving leg was held in extension during the whole movement, whereas the opposite thigh was held down on the bench by the physiotherapist. The angle (^◦^) between the bench and the femoral bone on the flexed leg was measured by a goniometer, with the pivot point at the trochanter major.

#### Questionnaire data

All participants answered a questionnaire including questions about age and experience of hamstring strain injuries during the last 2 years (yes/no). Based on the answer regarding hamstring strain injury, players were divided into two groups for the statistical analyses: players who had suffered from hamstring strain injury, and players who had not.

### Statistical analyses

Mean and *SD* was calculated for each variable. Visual inspection of histograms confirmed that the data were normally distributed. Independent sample *t*-tests were used to investigate any differences in physical performance between players who had and had not experienced hamstring strain injuries. Mixed between-within subject's analyses of variance, with one between group factor (presence and absence of hamstring strain injury the last 2 years) and one within subjects/repeated-measures factor (8 × 20 m sprints) were conducted to assess the effect of a former hamstring strain injury on the player's ability to perform repeated sprints. The interaction effects (group × time) are reported. When significant, an interaction effect indicates a different development over time between the groups. Intraclass correlation coefficient (ICC) analyses with a two-way mixed model and absolute agreement were performed to evaluate the reliability of the repeated sprint test. Typical error (SEM) was calculated as SD/√(1-ICC) (Hopkins, 2000). Minimal detectable change, reflecting the 95% confidence interval (MDC_95_), a statistical estimate of the smallest amount of change that can be detected by a measure that corresponds to a noticeable change in ability, was calculated as SEM √2 × 1.96. *Post-hoc* power calculations with two-tailed distribution and an alpha error level of 5% were performed for the various tests. Significance levels were set at <0.05. The Statistical Package for Social Science (SPSS Statistics, version 23) for Windows was used for all statistical analyses.

#### Ethics

The study followed the principles of the Helsinki declaration. All players signed a written informed consent form and were informed about the right to withdraw from the study at any time. Norwegian Social Science Data Services (NSD) approved the study.

## Results

Of the 75 players who performed the repeated sprint test, 16% had reported hamstring strain injury during the 2 previous years. An independent sample *t*-tests showed no significant difference in age, height, body weight, or body fat percentage between players who had and had not experienced hamstring strain injury during the previous 2 years (Table 1).

**Table 1:** **An overview of characteristics of the players**.

	**Former hamstring strain injury (*n* = 12)**	**No hamstring strain injury (*n* = 63)**	***p*****-values**
Age (years)	19 ± 2	18 ± 3	0.668
Hight (cm)	180 ± 6	180 ± 6	0.901
Weight (kg)	74 ± 5	73 ± 10	0.473
Body fat (%)	10.7 ± 3.0	10.0 ± 3.2	0.490

*Data is presented as mean ± standard deviation (SD). Between-group differences were evaluated by t-tests for independent samples*.

**Figure 3:**
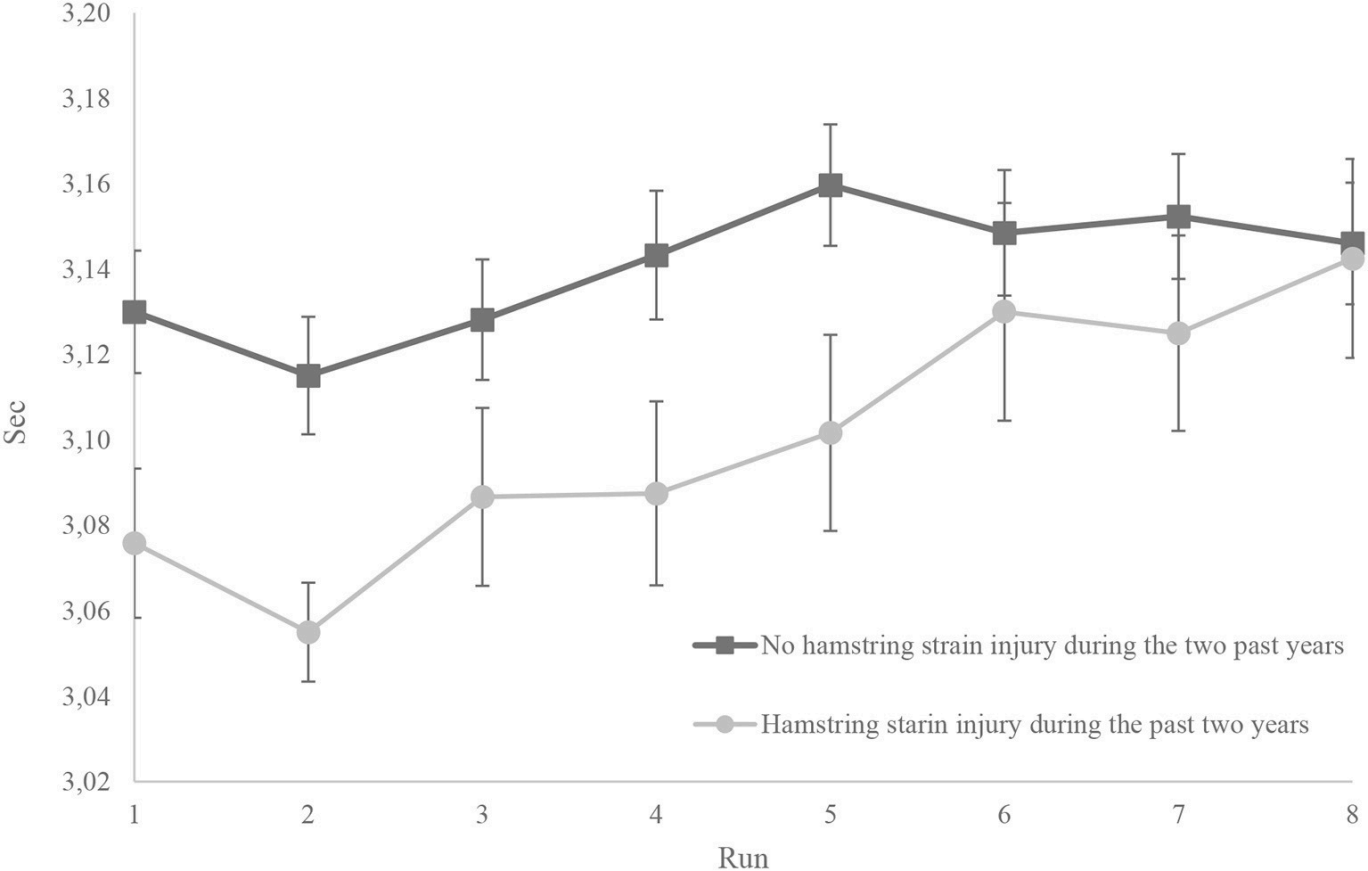
**An overview of mean time and standard error of the mean (vertical lines) for each 20 m run for players with and without former hamstring strain injury**.

Players reporting previous hamstring strain injuries showed a significantly higher drop in speed (i.e., mean of the last two runs minus mean of the first two runs) during the repeated sprint test compared to those without hamstring strain injuries during the previous 2 years (Table 2 and Figure 3). The mixed between-within subject's analyses of variance showed a significant interaction (group × time) (*F* = 3.22, *p* = 0.002), indicating that speed in the two groups changed differently during the repeated sprint test. The ICC for average measures was 0.978 (95% confidence interval: 0.969–0.985), SEM was 0.008 s, and MDC_95_ was ± 0.022 seconds. *Post-hoc* power analyses showed a statistical power of 88.8%.

**Table 2:** **An overview of results obtained in the 40 m linear sprint test, the 8 × 20 m repeated sprint test, countermovement jump, leg strength, VO_**2max**_ and in the hamstring flexibility tests in players with, and without experience of hamstring strain injury during the past 2 years**.

	**Former hamstring strain injury (*n* = 12)**	**No hamstring strain injury (*n* = 63)**	***p*****-values**
**LINEAR SPEED (s)**			
0–40 m	5.26 ± 0.09	5.35 ± 0.20	0.151
**8 × 20 m REPEATED SPRINT TEST (s)**			
Mean speed	3.10 ± 0.06	3.14 ± 0.10	0.222
Drop in speed^a^	0.07 ± 0.05	0.02 ± 0.05	0.007
**HAMSTRING FLEXIBILITY**			
Sit and reach test (cm)	27.8 ± 9.6	30.6 ± 8.8	0.319
Straight leg raise test right leg (^◦^)	74 ± 13	80 ± 13	0.159
Straight leg raise test left leg (^◦^)	75 ± 13	80 ± 14	0.305
VO_2max_(ml/kg/min)	63.2 ± 4.8^b^	63.6 ± 5.2	0.811
Countermovement jump (cm)	33.2 ± 4.0	33.0 ± 4.8^c^	0.902
5 RM squat (kg)	81 ± 10^d^	79 ± 16^e^	0.618

a Drop = [(mean time of the seventh and eight run) minus (mean time of the first and second run)];

b *n = 10*,

c *n = 62*,

d *n = 11*,

e *n = 59*.

*Data is presented as mean ± standard deviation (SD). Between-group differences were evaluated by independent sample t-tests*.

There was however, no significant difference in mean time of the eight runs between the two groups. There was also no significant difference in linear speed (statistical power of 70.1%), in hamstring flexibility (SLR test: statistical power of 15.5%), or in countermovement jump height (statistical power of 5.3%), the 5 RM squat test (statistical power of 8.5%) or in VO_2max_ (statistical power of 5.7%) between the two groups.

## Discussion

Our result showed that soccer players who reported previous hamstring strain injury (16%) had a significantly higher drop in speed during the repeated sprint test compared to players reporting no hamstring strain injury.

Repeated sprinting ability is very important to succeed in soccer (Ingebrigtsen et al., 2015). The higher drop in speed during the repeated sprint test in players with previous hamstring strain injury, may be related to neuromuscular fatigue and insufficient rehabilitation. A recent study by Padulo et al. (2016) showed increased neuromuscular fatigue, measured as reduced jumping height after repeated sprints in players with previous lower limb injuries. Increased neuromuscular fatigue was evident even though the players had been cleared to return to play by the team physician, and had completed a standard 3-month rehabilitation program and a standard 3-month soccer-training program (Padulo et al., 2016). There was no significant difference in CMJ height between groups after the repeated sprint test in the present study. This may be due to the use of different repeated sprint test with different lower-limb muscle load or to different time between the tests. The CMJ was performed immediately after the repeated sprint test in the study by Padulo and colleague, and ~1 h after in the present study.

Padulo and colleagues reported however no difference between players who had suffered from previous injury with regard to repeated sprint performance, which differs from our findings in the present study. This incongruence may be due to the use of different repeated sprint tests (6 × 40 m shuttle runs vs. 8 × 20 m), and different ways of evaluating the results of the tests (fatigue index vs. drop in speed). The muscular recruitment and the biomechanical demands of the hamstring muscles is different between sprints including acceleration, decelerations, and change of directions compared to sprints only including accelerations as in the present study. In addition, Padulo and colleagues included players with a variety of lower limb injuries, whereas we only included players with previous hamstring strain injuries. Although Padulo and colleagues found no significant difference in the repeated sprint performance, players with previous lower limb injuries reported a significantly higher rate of perceived exertion, indicating that they were more fatigued (Padulo et al., 2016).

Neuromuscular fatigue has been suggested to be a potential risk factor for muscle strain injury (Heiser et al., 1984; Worrell et al., 1991; Mair et al., 1996; Woods et al., 2004; Ekstrand et al., 2011b), indicating that insufficient rehabilitation after a hamstring strain injury may increase the risk of re-injury. Fatigue while playing a soccer match has been associated with decreased eccentric hamstring strength (Worrell et al., 1991) in healthy players. Another study has shown that intermittent running, designed to mimic the demands of competitive soccer, significantly reduces eccentric hamstring torque (Greig, 2008; Small et al., 2009). Mair et al. (1996) confirm this finding in a laboratory study, showing that pre-fatigue muscles absorbed less energy before failure compared to unfatigued muscles, indicating that a fatigued muscle may be more likely to suffer from a strain injury due to its reduced capacity to resist over-lengthening. It is claimed that those who exhibit greater levels of eccentric hamstring fatigue may be at a greater risk of hamstring strain injury, thus linking eccentric weakness and risk of hamstring injury (Croisier et al., 2008; Sugiura et al., 2008). The higher drop in speed during the repeated sprint test may be an indication of increased disposition to hamstring muscle fatigue in players with previous hamstring injury. Although neuromuscular fatigue is also found in healthy players after a soccer match, and after intermittent running designed to mimic the demands of competitive soccer, the identification of fatigue only after an 8 × 20 m repeated sprint test may indicate the need for targeted reconditioning programs to ensure complete post-injury rehabilitation in players recovering from hamstring strain injury.

Inappropriate hamstring muscle recruitment patterns (Schuermans et al., 2016) are suggested as factors linking neuromuscular fatigue with the elevated risk of strain injuries (Devlin, 2000). Schuermans et al. (2016) recently demonstrated a causal relationship between the intramuscular recruitment pattern and the risk of a secondary hamstring strain, indicating that intermuscular interplay changes after hamstring strain injury. Higher drop in speed in the repeated sprint test may be related to insufficient rehabilitation due to changed muscle recruitment pattern (Schuermans et al., 2016).

Previous studies have shown that the physiological and mechanical demands associated with league soccer games produce a time-dependent alteration in sprinting kinematics (Pinniger et al., 2000; Small et al., 2009, 2010). An inappropriate sprinting technique, *per se*, and a change in sprinting technique due to fatigue may both be risk factors for hamstring strain injury due to the increased eccentric hamstring muscle load. Studies have shown that technical changes due to fatigue cause greater strain on the hamstring muscle during the terminal swing phase of the gait (Pinniger et al., 2000; Thelen et al., 2005), with the consequence that repeated over-lengthening and microscopic muscle damage may accumulate to a hamstring strain injury (Morgan, 1990; Allen et al., 2010). The higher drop in speed in players with previous hamstring strain injury may be related to inappropriate sprinting technique or a change in sprinting technique during the repeated sprint test. Inspection of sprinting technique during rehabilitation is therefore essential.

Although information regarding previous hamstring strain injury was self-reported, the incidence rate of 16% in the present study is in line with that previously reported in comparable studies (Arnason et al., 2004; Woods et al., 2004; Ekstrand et al., 2011b). Despite lack of clinical information related to hamstring strain severity and location, our data showed significant differences regarding drop in speed of players reporting previous hamstring strain injury compared to players reporting no previous hamstring strain injury. Based on the above mention factors, we suggest that the drops in speed in the repeated sprint test are an important and novel characteristic of players with previous hamstring strain injury.

There was no significant difference in maximal speed, countermovement jump height, leg strength, endurance capacity, and hamstring flexibility between players who had and had not suffered from hamstring strain injury. Hence, the above mention factors do not seem to explain the significantly higher drop in speed in players with previous hamstring strain injury. However, we cannot conclude due to low statistical power. It would be interesting in a future study also to evaluate potential asymmetry between a previously injured leg and an uninjured leg in strength, and to evaluate possible asymmetries between knee flexors and extensors (Dello Iacono et al., 2016).

## Conclusion

A significantly higher drop in speed during repeated sprints was observed in soccer players who reported previous hamstring strain injury compared to players who did not. A repeated sprint test consisting of 8 × 20 m could be used as a field-based diagnostic tool to identify players who are in need of targeted reconditioning programs to ensure complete post-injury rehabilitation.

## Author contributions

OR conceptualized and designed the study, took part in data collection, drafted the initial manuscript, and approved the final manuscript as submitted. HG took part in conceptualization and design of the study. Did all the statistical analyzes, drafted the manuscript for important intellectual content, and approved the final manuscript as submitted. The coauthors MK, BB, AW, ME, AN, VI, and SM have all participated in conception of this manuscript, collection of data and have assisted in revising the manuscript for important content. All authors have provided final approval of the enclosed manuscript.

## Funding

This work was funded by Bergen University College, Postboks 7030, 5020 Bergen, Norway.

### Conflict of interest statement

The authors declare that the research was conducted in the absence of any commercial or financial relationships that could be construed as a potential conflict of interest.
